# Resistance Training Regulates Cardiac Function through Modulation of miRNA-214

**DOI:** 10.3390/ijms16046855

**Published:** 2015-03-26

**Authors:** Stéphano Freitas Soares Melo, Valério Garrone Barauna, Miguel Araújo Carneiro Júnior, Luiz Henrique Marchesi Bozi, Lucas Rios Drummond, Antônio José Natali, Edilamar Menezes de Oliveira

**Affiliations:** 1 Laboratory of Biochemistry and Molecular Biology of the Exercise, School of Physical Education and Sport, University of Sao Paulo, Sao Paulo 05508-030, Brazil; E-Mails: stephanomelo@usp.br (S.F.S.M.); luizbozi@hotmail.com (L.H.M.B.); 2 Laboratory of Molecular Physiology, Health Sciences Center, Federal University of Espírito Santo, Vitória 29043-900, Brazil; E-Mail: barauna2@gmail.com; 3 Department of Physical Education, Federal University of Viçosa, Viçosa, Minas Gerais 36570-900, Brazil; E-Mails: miguelefiufv@yahoo.com.br (M.A.C.J.); lucasriosufv@yahoo.com.br (L.R.D.); anatali@ufv.br (A.J.N.)

**Keywords:** resistance training, cardiovascular, microRNA, cardiomyocytes

## Abstract

Aims: To determine the effects of resistance training (RT) on the expression of microRNA (miRNA)-214 and its target in sarcoplasmic reticulum Ca^2+^-ATPase (SERCA2a), and on the morphological and mechanical properties of isolated left ventricular myocytes. Main methods: Male Wistar rats were divided into two groups (*n* = 7/group): Control (CO) or trained (TR). The exercise-training protocol consisted of: 4 × 12 bouts, 5×/week during 8 weeks, with 80% of one repetition maximum. Key findings: RT increased the left ventricular myocyte width by 15% and volume by 12%, compared with control animals (*p* < 0.05). The time to half relaxation and time to peak were 8.4% and 4.4% lower, respectively, in cells from TR group as compared to CO group (*p* < 0.05). RT decreased miRNA-214 level by 18.5% while its target SERCA2a expression were 18.5% higher (*p* < 0.05). Significance: Our findings showed that RT increases single left ventricular myocyte dimensions and also leads to faster cell contraction and relaxation. These mechanical adaptations may be related to the augmented expression of SERCA2a which, in turn, may be associated with the epigenetic modification of decreased miRNA-214 expression.

## 1. Introduction

Several studies suggest that resistance training (RT) has beneficial effects on the cardiovascular system and can potentially be an effective treatment for various clinical conditions as such heart disease [1,2,3,4,5]. However, the molecular cardiovascular adaptations induced by RT are not as well described as those induced by aerobic training. We and others have previously demonstrated that RT may lead to a physiological type of concentric cardiac hypertrophy, and some molecular and cellular adaptations have been described [4,5,6,7].

Aerobic exercise is characterized by the use of large muscle groups in dynamic physical activities, such as running and swimming. It increases cardiac preload and is known to induce the eccentric type of cardiac hypertrophy, which manifests as increased left ventricular (LV) cavity dimensions and proportionally augmented LV wall thickness to normalize myocardial strain [8,9,10,11]. At the ultrastructural level, new sarcomeres added in series predominate resulting in increased cardiomyocyte length [12,13,14,15,16].

On the other hand, the strength or resistance type of exercise, which increases cardiac after load due to increased peripheral vascular resistance, leads to concentric cardiac hypertrophy that differs from that induced by aerobic exercise. This physiological concentric LV hypertrophy is characterized by increased LV wall thickness and no changes in the LV cavity dimensions [7,17]. This type of hypertrophy induces the addition of new sarcomeres in parallel, which results in increased cardiomyocyte width [7,18,19]. However, this hypothesis has not yet been investigated in isolated cardiomyocytes after RT.

The LV remodeling after aerobic exercise training is well known to improve ventricular function in both healthy and disease conditions [8,10] which has been demonstrated both in isolated papillary muscle as well as in isolated cardiomyocytes from animal models [15,20,21]. The improvement in cell function by aerobic exercise training is accompanied by an increase in Ca^2+^ uptake by the sarcoplasmic reticulum Ca^2+^-ATPase (SERCA2a) that actively transports Ca^2+^ into the cardiac sarcoplasmic reticulum, regulates cytosolic Ca^2+^ concentration and plays a pivotal role in myocardial contractility [15,22]. This intrinsic contractility adaptation of cardiomyocytes is the key mechanism to explain the improved myocardial contractile function induced by exercise.

MicroRNA (miRNAs) are small (approximately 22 nucleotides) single-stranded non-coding RNAs that are transcribed into the nucleus, processed by specific enzymes and incorporated in RNA-induced silencing complexes (RISC), which inhibit the transcription of the target mRNA and its translational processing into the mature protein. We have recently described and reviewed some of the miRNAs involved in the physiological cardiac hypertrophy induced by exercise training [23,24,25,26].

Thus, since SERCA2a is an interesting target involved on cardiomyocyte function and is modulated by miRNA-214, we sought to determine the effects of RT on miRNA-214 expression and its target protein SERCA2a, and on the morphological and mechanical properties of isolated LV myocytes.

## 2. Results

### 2.1. Body Mass and Left Ventricular Mass

No difference was observed between groups for body weight (BW) during the study period. Cardiac hypertrophy analyzed by LV weight/BW ratio was 21.7% greater in the trained (TR) group compared with the control (CO) group (Table 1).

**Table 1:** Body weight (BW), left ventricular (LV), left ventricular weight/body weight ratio of 7 animals per control (CO) and trained (TR) groups. Results are presented as mean ± standard deviation. * *p* < 0.05 when compared with the control group. One repetition maximum (1RM).

Parameters	CO	TR
BW (g)	348.9 ± 6.7	341.7 ± 8.5
LVW (mg)	632.1 ± 30.9	753.5 ± 35.4
LV/BW (mg/g)	1.8 ± 0.06	2.2 ± 0.04 *
1RM Initial (g)	639.2 ± 35.2	643.7 ± 41.4
1RM Final (g)	1070.4 ± 41.7	2168.2 ± 37.8 *

### 2.2. Maximal Strength

Analysis of the 1 repetition maximum (1RM) test showed an increase in the absolute weight lifted by the TR group obtained during the 1RM test. Both the CO and the TR group had similar values for 1RM at the beginning (day 0) of the protocol. After the 8-weeks of RT protocol, the load lifted by the TR group was higher (2168 ± 37 g) than that of the control (1070 ± 41 g) group, which represented a 2-fold increase for the TR group (Table 1).

### 2.3. Effects of Training on Cardiomyocyte Dimensions

**Figure 1:**
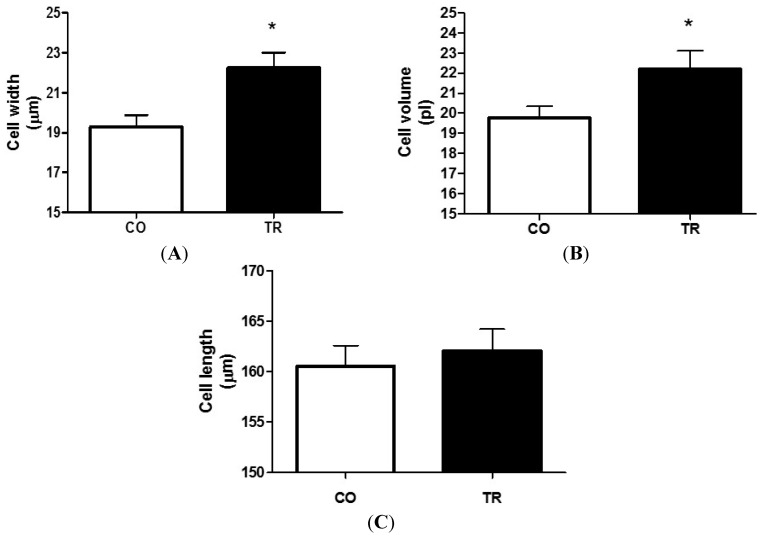
Cardiomyocyte dimensions of isolated LV myocytes. (**A**) Cell width; (**B**) Cell volume; and (**C**) Cell length. Results are presented as mean ± standard deviation of 70 cells per group and 7 animals each group. At least 10 cells were analyzed from each animal. * *p* < 0.05 when compared to control group.

Figure 1 shows that RT increased LV myocyte width by 15.3% (Figure 1A) and volume by 12.2% (Figure 1B) respectively, in the TR group when compared to the CO group. However, cell length was not changed by RT (Figure 1C, *n* = 7 rats and 70 cells per group).

### 2.4. Cell Contractility

**Figure 2:**
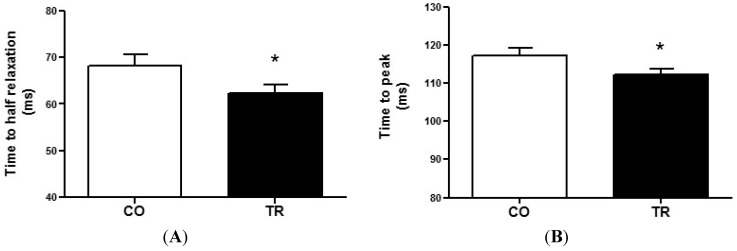
Cell contractility (**A**) Time to half relaxation; and (**B**) Time to peak contraction of cardiomyocytes from trained (*n* = 7 rats and 108 cells) and control (*n* = 7 rats and 96 cells) groups. Results are presented as mean ± standard deviation. * *p* < 0.05 when compared to control group.

To measure cardiomyocyte contractility, isolated LV cells were used. The time to half relaxation (Figure 2A) and time to peak (Figure 2B) were 8.4% and 4.4% lower, respectively, in cells from TR group (*n* = 7 rats and 108 cells) compared to those from CO group (*n* = 7 rats and 96 cells). These data demonstrate that resistance trained animals exhibit faster cell contraction and relaxation.

### 2.5. Protein and miRNA Expression

**Figure 3:**
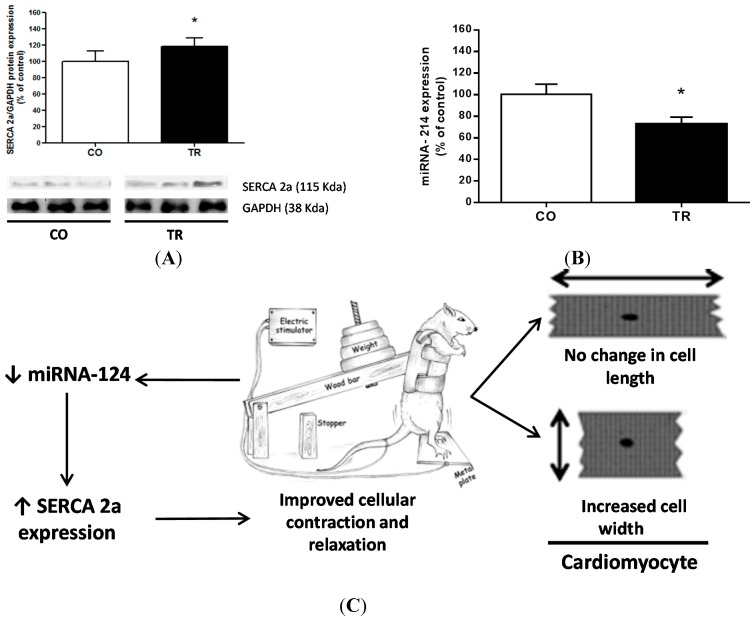
Protein expression level and miRNA. (**A**) Representative western blot image and SERCA2a protein expression; (**B**) miRNA expression level; and (**C**) Schematic summary of the results showing both the morphological and functional adaptations induced by the resistance training. Results are presented as mean ± standard deviation. * *p* < 0.05 when compared to control group.

To further understand the molecular mechanisms that could explain the functional data on isolated LV myocytes, SERCA2a protein expression, a key molecule involved in Ca^2+^ sequestration into the sarcoplasmic reticulum, was analyzed. SERCA2a expression levels were 18.5% higher in the TR group than in CO group (Figure 3B), which may be explained by the 18.5% decrease in the miRNA-214 level (Figure 3A).

## 3. Discussion

The present study provides the first observations regarding the effects of RT on morphological and mechanical properties of single LV myocytes. RT increased width and volume of isolated LV myocytes. Also, exercised animals exhibited faster cell contraction and relaxation, which can be explained by the decreased miRNA-214 expression and increased SERCA2a protein levels.

In study involving humans, Fagard (1996) [11] observed that athletes engaged in static training had LV dimension increased by 2.5% and LV wall thickness increased by 12% compared with non-athletes. In addition, Dickerman *et al.* (1998) [27] demonstrated that a LV wall thickness greater than 13 mm can routinely be found in elite resistance-trained athletes. Previous data from our group has confirmed this data in the animal model used here showing that RT induces concentric cardiac hypertrophy. Echocardiography analysis in this animal model of RT showed a similar increase in both septum and free posterior wall mass, but no reduction in the end-diastolic LV internal diameter during 3 months of exercise training [28].

Cardiomyocytes isolated from aerobic exercise-trained rats showed increased cell length [20,29,30], with no change in thickness [13,31,32]. At the same time, in other studies, it was observed that the intermittent type of aerobic exercise training increased the thickness of LV myocytes in rats with no change in their length. It was also observed that this adaptation was more evident in cardiomyocytes from the region near to the endocardium [14,21]. Here we are showing that RT, differently from the aerobic exercise training, resulted in increased width and volume of LV myocytes when compared with sedentary control animals, while cell length was unaltered. Thus, the results show that due to the increased cardiac after load during the RT exercise sessions, cardiac cell width and volume were also increased which are in agreement with previous echocardiographic data from our group [28].

In addition to these morphological adaptations, we also observed that single cardiomyocytes had improved contraction and relaxation function. Pinter *et al.* (2008) [5], showed increased myosin ATPase activity associated with increased papillary muscle contractility after 8 weeks of RT. The papillary muscles of exercised rats showed increased isometric force, indicating that the RT might improve cardiac performance. In humans, data are controversial. Echocardiography results have demonstrated that cardiac function is not altered in RT athletes [18,33], although other studies have shown enhanced systolic function [9,34] and one study indicated enhanced diastolic function [35]. However, with respect to the results observed by echocardiography, although it is a measure of the cardiac function in the whole organ, it is necessary to consider that this involves a large number of mathematical calculations while the direct cellular measurements in isolated myocytes are more reliable.

Aerobic exercise training improves cardiomyocyte contractility and Ca^2+^ handling [31,32], which optimizes cardiac performance [36,37]. Cardiac myocyte shortening and relaxation kinetics are regulated by Ca^2+^ regulatory proteins, contractile protein isoform expression patterns, and different action potential waveform and duration [22,38]. Although we did not analyze all these possible mechanisms, the increase in SERCA2a protein expression, which is responsible for 92% of Ca^2+^ reuptake in rat ventricular cells [37] may partly explain our findings. The time to peak and time to half relaxation were lower in cells of the TR group. Our findings might also be explained by recent studies suggesting that mice with overexpression of the SERCA2a pump exhibit increased sarcoplasmic reticulum Ca^2+^ transport, which in turn increases the rates of cardiac contraction and relaxation without developing cardiac pathology [39,40,41,42]. Furthermore, a possible role for other important modulators of SERCA2a, such as its ATPase activity and the phospholamban phosphorylation, which regulates SERCA2a function, must be considered as well [43].

miRNAs have been implicated in regulating the expression of genes that are involved in multiple biological processes of cardiovascular disease and also as potential drug targets[44]. Recently, Gurha *et al.* (2012) [45], showed that genetic ablation of miRNA-22 regulates target proteins that function as transcription factors for SERCA2a expression, and Wahlquist *et al.* (2014) [46], showed that miRNA-25 regulates SERCA2a and contributes to declining cardiac function during heart failure. On the other hand, Aurora *et al.* (2012) [47], reported that miRNA-214 targets both sodium/calcium exchanger 1 (NCX) and proapoptotic effectors of Ca^2+^-signaling pathways like CaMKII and cyclophilin D. Through *in silico* analysis of predicted targets for miRNA, we verified a possible relationship between SERCA2a and miRNA-214. Here, our results showed that decreased miRNA-214 levels in the trained group may explain the increased expression of SERCA2a. This relationship becomes of great interest because our results show that the regulation of SERCA2a by miRNA-214 occurs by exercise training. Such regulation is crucial in as much as SERCA2a represents 90% of the total membrane proteins in the sarcoplasmic reticulum of the rat myocardium and has a massive impact on cardiac contractile function.

The SERCA2a isoform is also found in skeletal muscle and is modulated by aerobic exercise training. In this context, SERCA2a expression has been shown to be modulated by the adenosine monophosphate-activated protein kinase (AMPK)-α_2_ in type I skeletal muscle fibers [48] and by adiponectin [49], two other possible pathways that were not investigated here.

## 4. Methods

### 4.1. Animals

All procedures were in accordance with the Brazilian Society for Laboratory Animal Science (COBEA) and were approved by the Ethics Committee of the School of Physical Education and Sport of the University of São Paulo (Process Number: 2009/34, Date of Approval: 9 April 2009). Fourteen male Wistar rats (250–300 g and 10-weeks-old) were randomly divided into two groups (*n* = 7): Control (CO) and trained (TR). Animals were housed in standard cages, with food and water *ad libitum.* The environmental temperature was kept at 23 ± 1 °C, and a 12:12-h dark-light cycle was maintained throughout the experiment.

### 4.2. Exercise Training Protocol

**Figure 4:**
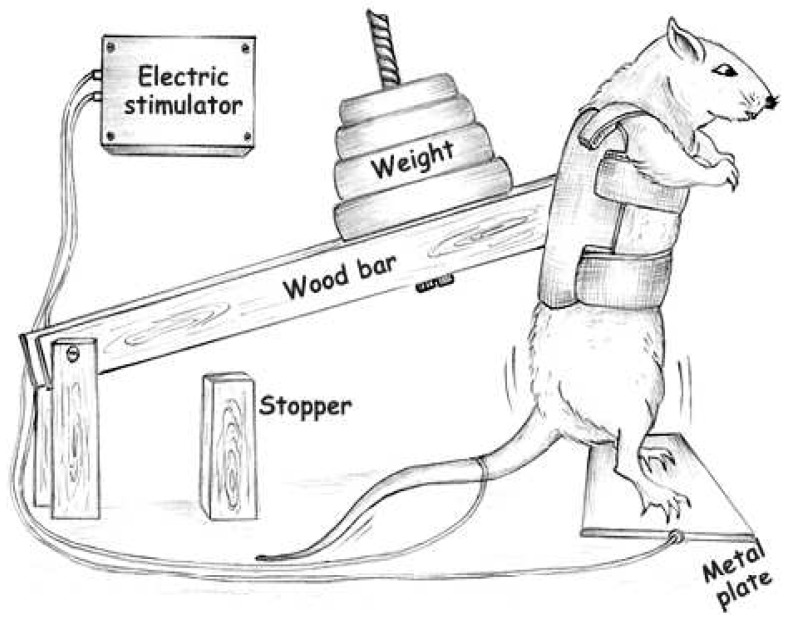
Apparatus adapted from Tamaki *et al.* (1992) [50], used to perform resistance training in the rats [3,4].

Animals were trained following previous studies by our group [3,4] (Figure 4) and adapted from Tamaki *et al.* (1992) [50]. Briefly, rats were wearing canvas jackets to be able to regulate the twisting and flexion of their torsos and were suspended in a standard position on their hindlimbs. Electrical stimulation (20 V, 0.3-s duration, at 3-s intervals) was applied to the tail through a surface electrode. Stimulated rats flexed their legs repeatedly, which lifted the weight arm of the training apparatus. The rats were trained by 4 × 12 repetitions, with a 90-s rest period between each set for eight weeks. The animals were adapted for one week and on the last day of adaptation, the maximum weight lifted [one repetition maximum (1RM)] was measured with the squat-training apparatus, and the training load was set at 80% of this value. The 1RM value was defined as the minimum load in which the rats were unable to jump after electrical stimulation. Exercise training sessions were performed in a dark room.

### 4.3. Cardiomyocytes Isolation

Twenty-four hours after the last 1RM test, the rats were killed by decapitation under resting conditions, and their hearts were quickly removed and weighed. Ventricular cardiomyocytes were enzymatically isolated as previously described [14,16]. Briefly, the hearts were mounted on a home-made Langendorff system and perfused for 5 min with a modified Hepes–Tyrode’s solution of the following composition (in mM): 130 NaCl, 1.43 MgCl_2_, 5.4 KCl, 0.75 CaCl_2_, 5.0 Hepes [4-(2-hydroxyethyl)-1-piperazineethanesulfonic acid], 10.0 glucose, 20.0 taurine, and 10.0 creatine, pH 7.4 at 37 °C. The perfusion solution was changed for the calcium-free solution containing 0.1 mM EGTA (Ethylene glycol-bis(β-aminoethyl ether)-*N*,*N*,*N*',*N*'-tetraacetic acid) for 6 min. Afterwards, the hearts were perfused for 15–20 min with a solution containing 1 mg/mL collagenase type II (Worthington, Cooper Biomedical Co., Freehold, NJ, USA). The digested heart was then removed from the cannula, the LV was dissected and cut into small pieces. The left ventricle tissues were placed into small conical flasks with collagenase-containing solution supplemented with 1% bovine serum albumin (Sigma Chemicals, St. Louis, MO, USA). The cells were dispersed by shaking the flasks at 37 °C for periods of 5 min. Then, single cells were separated from the non-dispersed tissue by filtration. The resulting cell suspension was centrifuged and resuspended in Hepes–Tyrode’s solution. Non-dispersed tissue was subjected to further enzyme treatment. The isolated cells were stored at 5 °C until use. Only Ca^2+^ tolerant, quiescent, rod-shaped myocytes showing clear cross striations were studied. The isolated cardiomyocytes were used within 2–3 h after isolation.

### 4.4. Measurements of Cell Contractility and Morphology

Cell contractility was evaluated as previously described [16]. Briefly, isolated cells were placed in a chamber with a glass coverslip base mounted on the stage of an inverted microscope (Nikon Eclipse TS100, Nikon, Kawasaki, Japan). The chamber was perfused with Hepes-Tyrode’s solution at room temperature [16]. Steady-state 1-Hz contractions were elicited via platinum bath electrodes (MyoPacer field stimulator; IonOptix, Milton, MA, USA) with 5-ms-duration voltage pulses and an intensity of 20 V. Cells were visualized on a personal computer monitor with a NTSC camera (MyoCam, IonOptix, Milton, MA, USA) in partial scanning mode. This image was used to measure cell shortening (our index of contractility) in response to electrical stimulation using a video motion edge detector (IonWizard, IonOptix). The cell image was sampled at 240 Hz. Cell shortening was calculated from the output of the edge detector using an IonWizard analog-to-digital converter (IonOptix). Time to peak of contraction and time to half-relaxation was calculated as previously described [16]. The cell image was also used to determine cell lengths and widths, which were used to calculate the cell volume as previously described [21]. Measurements were performed in at least 10 cells from each animal, and in 7 animals from each group. The total numbers of cells analyzed are described in the legend of each figure.

### 4.5. Western Blot Analysis

Frozen ventricles were thawed and minced into small pieces and homogenized in cell lysis buffer containing 100 mM Tris, 50 mM NaCl, 10 mM EDTA, 1% Triton X-100, and a mixture of protease inhibitors [phenylmethanesulfonyl fluoride (1 mM) *o*-phenanthroline (30 mM), pepstatin A (1 mM) and 4-(chloromercuribenzoic acid) (1 mM)]. The heart debris tissues were removed by centrifugation at 3000× *g*, 4 °C, for 10 min. A total of 20 µg of protein per sample was loaded and subjected to SDS-PAGE gels. After electrophoresis, proteins were electro transferred to nitrocellulose membrane (Amersham Biosciences; Piscataway, NJ, USA). The blot membrane was incubated in a blocking buffer (5% nonfat dry milk, 10 mM Tris-HCl, pH 7.6, 150 mM NaCl, and 0.1% Tween 20) for 2 h at room temperature and probed with a polyclonal antibody directed against SERCA2a (1:2500; Abcam, Cambridge, UK) and a polyclonal anti-GAPDH antibody (1:2000; Abcam). The level of expression of GAPDH was used to normalize the results. Binding of the primary antibody was detected with the use of peroxidase-conjugated secondary antibodies, and enhanced chemiluminescence reagents (Amersham Biosciences; Piscataway, NJ, USA) were used to visualize the autoradiogram, which was later exposed to film. The film was developed, and the bands were analyzed using Scion Image software (Scion based on National Institutes of Health image). Total GAPDH protein expression was used to normalize the results. Protein content was determined using the protein assay by Bradford method (Bio-Rad, Richmond, CA, USA) and BSA (0.1–1 mg/mL) as standard.

### 4.6. miRNA Quantification Using Real-Time PCR

The relative expression of miRNA-214 and U6 were analyzed by polymerase chain reaction (PCR), described as follows. cDNA for miRNA analysis was synthesized from total RNA using gene-specific primers according to the TaqMan MicroRNA Assay protocol (Applied Biosystems, Foster City, CA, USA). In order to accurately detect the expression of miRNAs, real-time PCR quantification was performed using TaqMan MicroRNA Assay protocol (Applied Biosystems). TaqMan MicroRNA Assay protocol for miRNA-214 (ID000517). Samples were normalized by evaluating U6 (ID4373381) expression. Relative quantities of target gene expressions were compared after normalization to the values of reference gene (Δ*C*_t_). Fold changes in miRNA expression were calculated using the differences in Δ*C*_t_ values between the two samples (ΔΔ*C*_t_) and equation 2^−ΔΔ*C*t^. The results were expressed as % of control.

### 4.7. Statistical Analysis

Differences between groups were assessed using unpaired *t*-tests. The initial and final 1RM was assessed using paired *t*-test. Results are presented as mean ± standard deviation (SD). Data were significant when *p* < 0.05 compared with the control group.

## 5. Conclusions

We have shown that RT increased the width and volume of LV myocytes. In addition, we have observed that trained animals exhibited faster cardiomyocyte contraction and relaxation, and that this adaptation is at least partly explained by improved SERCA2a expression and decrease of miRNA-214 with RT. The morphological and mechanical cellular adaptations reported here may contribute to our understanding of mechanisms involved in cardiac hypertrophy and contractile activity in the heart as a result of RT. In summary our results suggest that the RT induces cardiac hypertrophy with improved contractile function of isolated cardiomyocytes.
